# Validation of the Vancouver Symptom Score Questionnaire for bladder and bowel dysfunction for Brazilian children and adolescents

**DOI:** 10.1590/S1677-5538.IBJU.2022.0495

**Published:** 2022-11-20

**Authors:** Fernanda Nunes Coelho Siqueira Pinto, José de Bessa, José Murillo Bastos, Gláucia Cristina Medeiros Dias, Mônica Maria de Almeida Vasconcelos, Eleonora Moreira Lima, Tailly de Souza Almeida, Ana Cristina Simões e Silva, Flávia Cristina de Carvalho Mrad

**Affiliations:** 1 Universidade Federal de Minas Gerais Faculdade de Medicina Unidade de Nefrologia Pediátrica Belo Horizonte MG Brasil Departamento de Pediatria, Unidade de Nefrologia Pediátrica, Faculdade de Medicina, Universidade Federal de Minas Gerais (UFMG), Belo Horizonte, MG, Brasil; 2 Universidade Estadual de Feira de Santana Departamento de Urologia Feira de Santana BA Brasil Departamento de Urologia, Universidade Estadual de Feira de Santana, Feira de Santana, BA, Brasil; 3 Universidade Federal de Juiz de Fora Faculdade de Medicina Departamento de Urologia Juiz de Fora MG Brasil Departamento de Urologia, Faculdade de Medicina, Universidade Federal de Juiz de Fora (UFJF), Juiz de Fora, MG, Brasil; 4 Faculdade de Ciências Médicas de Juiz de Fora e Maternidade Therezinha de Jesus Departamento de Urologia Juiz de Fora MG Brasil Departamento de Urologia, Faculdade de Ciências Médicas de Juiz de Fora e Maternidade Therezinha de Jesus, Juiz de Fora, MG, Brasil; 5 Universidade Federal de Minas Gerais Laboratório Interdisciplinar de Investigação Médica Belo Horizonte MG Brasil Laboratório Interdisciplinar de Investigação Médica, Universidade Federal de Minas Gerais (UFMG), Belo Horizonte, MG, Brasil

**Keywords:** Urinary Bladder, Neurogenic, Surveys and Questionnaires

## Abstract

**Objective:**

This study aimed to translate, and perform a cross-cultural adaptation, and validation of the Vancouver Symptom Score (VSS) for bladder and bowel dysfunction (BBD) for Brazilian children and adolescents

**Materials and Methods:**

Six steps were performed for the translation and cross-cultural adaptation: (1) translation, (2) synthesis of translations, (3) back-translation, (4) pre-final version of the translated instrument, (5) pilot test and degree of comprehensibility and (6) elaboration of the Brazilian version of the VSS. For validation, the Brazilian Dysfunctional Voiding Score (DVSS) questionnaire was used.

**Results:**

Validation was performed on a sample of 107 children and adolescents with a mean age of 9.2 ± 2.84 years, presenting BBD and 107 without BBD (control group-CG). There was a positive correlation (r = 0.91, 95% CI 0.88 to 0.93, p < 0.0001) between total VSS score and total DVSS score. VSS was higher in patients with BBD (p < 0.0001). The internal consistency estimated by Cronbach's alpha was 0.87 for patients with BBD. The VSS showed excellent diagnostic accuracy in detecting cases, with an area under the ROC curve of 98% (95% CI 0.96 to 0.99, p < 0.001). A cut-off value of >11 points produced a sensitivity of 100% (95% CI 96.4% to 100%) and a specificity of 91.8% (95% CI 85.1% to 95.6%).

**Conclusion:**

The translated, cross-culturally adapted, and validated VSS for the Brazilian population is a reliable and valid tool to identify symptoms of BBD in children and adolescents aged five to 16 years, whose first language is Brazilian Portuguese.

## INTRODUCTION

Bladder bowel dysfunction (BBD) comprises lower urinary tract dysfunction (LUTD) and bowel dysfunction (1–3). It represents approximately 40% of patients seen in pediatric urology clinics (3, 4). BBD can be associated with vesicoureteral reflux and recurrent urinary tract infections sometimes leading to renal scarring (5). Symptoms affect the quality of life of children and adolescents, resulting in low self-esteem, social isolation and can lead to impaired learning (6).

Early recognition and non-invasive management of BBD in children and adolescents in a general outpatient setting plays an important role in reducing morbidity (5, 7). Several validated voiding scales are useful for this purpose (3, 5), including the Dysfunctional Voiding Symptom Score (DVSS) (8) and the Vancouver Symptom Score (VSS) (9). Scored questionnaires help diagnosis, allow measurement of severity, and monitor the effectiveness of treatment (3, 5, 10).

The VSS is an instrument that has undergone a careful validation process in the original development phase and uses appropriate measurement properties. It is a short questionnaire that addresses BBD in children and adolescents aged four to 16 years old, with a well-established cut-off value indicating its diagnostic accuracy. The VSS proved to be a valid and reliable tool for identifying BBD in children and adolescents and can be used both in clinical practice and research (9, 11). Drzewiecki et al. showed that VSS could be a diagnostic support tool for BBD and a way to assess treatment effectiveness (12).

Although the translated and cross-culturally adapted version of the DVSS questionnaire for the Brazilian population (13) has proved to be a valuable tool for screening and diagnosing BBD, it does not contain questions about enuresis if compared to VSS. In addition, the way in which the intensity of the investigated symptoms is graded presents great difficulty in application and interpretation. Besides, recently, the translation and cross-cultural adaptation of the Childhood Bladder and Bowel Dysfunction Questionnaire (CBBDQ) was carried out, which will allow the quantitative assessment of BBD in Brazilian children, but it can only be applied up to 12 years of age and a cutoff point was not yet defined for diagnostic purposes (14).

Using the DVSS and VSS instruments, we hypothesized that patients with BBD would have higher VSS scores than children and adolescents without BBD. In addition, patients with higher VSS scores would also have increased DVSS scores. Therefore, in this context, the objective of the present study was to carry out the process of translation, cross-cultural adaptation, and validation of the English version of the VSS for safe use in clinical practice and scientific research in the Brazilian population.

## MATERIALS AND METHODS

### Ethical approval

The institution's Ethics Committee approved the study under protocol CAAE 39015220.0.0000.5149, position statement 4.487.157. Legal guardians and participants aged 10 and 16 years signed the Informed Consent Term and the Assent Term, respectively. The corresponding author of the original study authorized the translation, cross-cultural adaptation, and validation of the VSS for the Brazilian population.

### Instrument

#### Vancouver Symptom Score (VSS)

**Figure 1:**
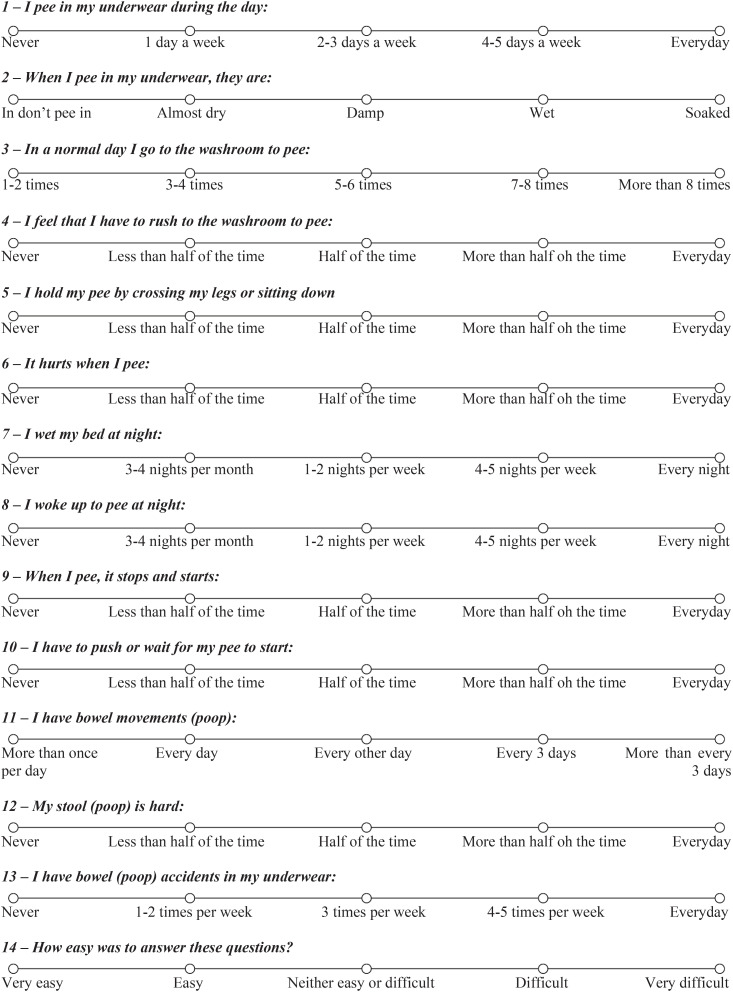
Vancouver Symptom Score (VSS) for Bladder Bowel Dysfunction adapted from Afshar et al. 2009 (9).

The VSS instrument was built as a self-administered questionnaire. All items were weighted equally. The questionnaire has 13-item condition-specific measures to assess symptoms of BBD (ten of bladder symptoms and three of bowel symptoms). A five-point Likert scale is used for all questions. Each question refers to a single symptom. A score of zero represents no complaints, while a score of four indicates severe symptoms. Only the question three about voiding frequency is scored different from other questions to establish voiding frequency abnormalities. The neutral choice (five to six urination per day) scored zero. Urinary frequency of one to two times or more than eight times a day has a score of four. Urinary frequency of three or four and seven or eight times a day corresponds to a score of two. The instrument total score ranges from zero to 52, being higher scores indicative of more severe symptoms. A total score of 11 was associated with 80% sensitivity and 91% specificity for symptoms of BBD. There is still one more question (item 14) that addresses feedback regarding the facility or not in completing the questionnaire and is not included in the total score (9) (Figure-1).

### Study design

The study was conducted following a biphasic validation methodology.

Stage 1: The translation into Brazilian Portuguese and the cross-cultural adaptation for the Brazilian population (15–18).

Stage 2: Validation of the translated and cross-culturally adapted instrument in a sample of Brazilian children and adolescents (15, 18–21).

**Figure 2:**
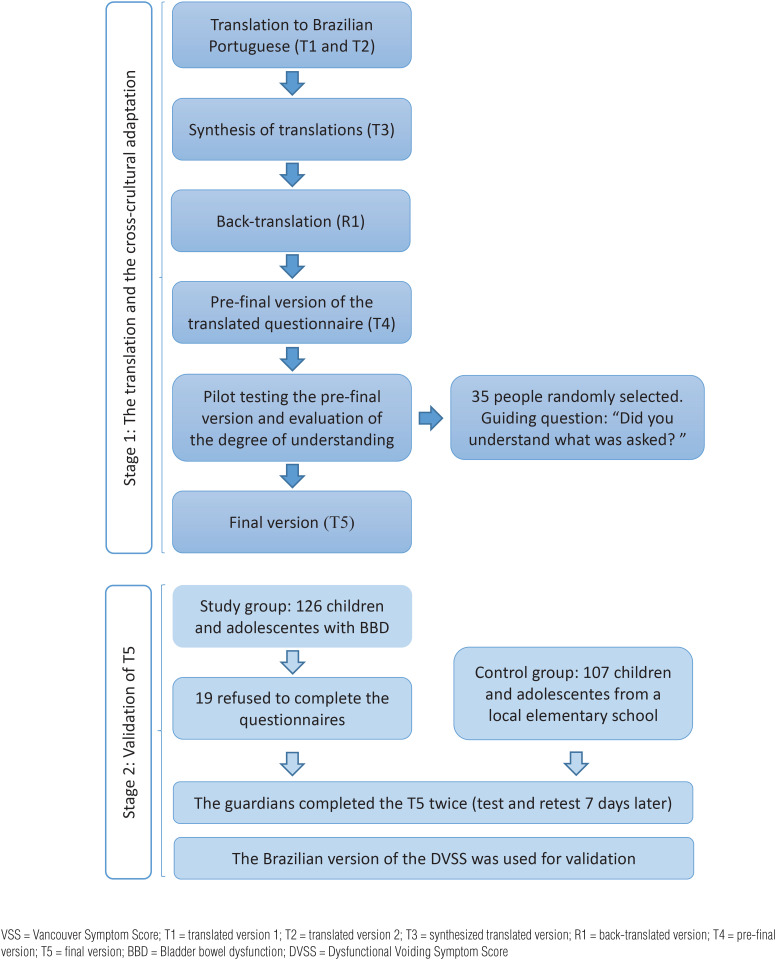
Flowchart with the steps involved in the translation, cross-cultural adaptation, and validation of the Vancouver Symptom Score (VSS) (15–21).

Flowchart with the steps involved in the translation, cross-cultural adaptation, and validation of the VSS questionnaire is shown in Figure-2.

#### Stage 1: The translation and the cross-cultural adaptation (15–18)

A group of eight health professionals, composed of physicians and a physical therapist with extensive experience in pediatric urology participated in this six-phase stage.

#### Phase 1: The translation for Brazilian Portuguese

Two physicians, whose native language is Brazilian Portuguese and who were fluent in English, independently translated the original VSS questionnaire into Brazilian Portuguese. Two translated versions (T) of the questionnaire were generated: T1 and T2.

#### Phase 2: Synthesis of translations

A meeting was held between the two translators who participated in Phase 1 and the team of experts. This group of professionals produced the synthesized direct translation nominated T3 based on the evaluation, reflection, and discussion.

#### Phase 3: Back-translation

The T3 was then independently back-translated (R1) into English by a bilingual translator. This translator did not participate in the first phase, was not a health professional, and was not informed about the concepts explored by the instrument. The translation was performed without prior knowledge of the original version of the questionnaire.

#### Phase 4: Pre-final version of the translated questionnaire

The committee of experts analyzed the versions generated in the previous stages (T1, T2, T3 and R1) and compared them with the original questionnaire. After consensus, the translated versions were edited and consolidated in the joint development of the pre-final version of the VSS questionnaire for Brazilian Portuguese nominated T4.

#### Phase 5: Pilot testing the pre-final version and evaluation of the degree of understanding

According to the protocol described by Beaton et al. (15), the primary researcher applied the pre-final version (T4) to 35 people randomly selected from different age groups and educational levels. The guiding question for the evaluation of the T4 version was: “Did you understand what was asked?” with the answer being YES or NO. Participants could request the researcher's mediation in case of difficulty.

#### Phase 6: Final version

**Figure 3:**
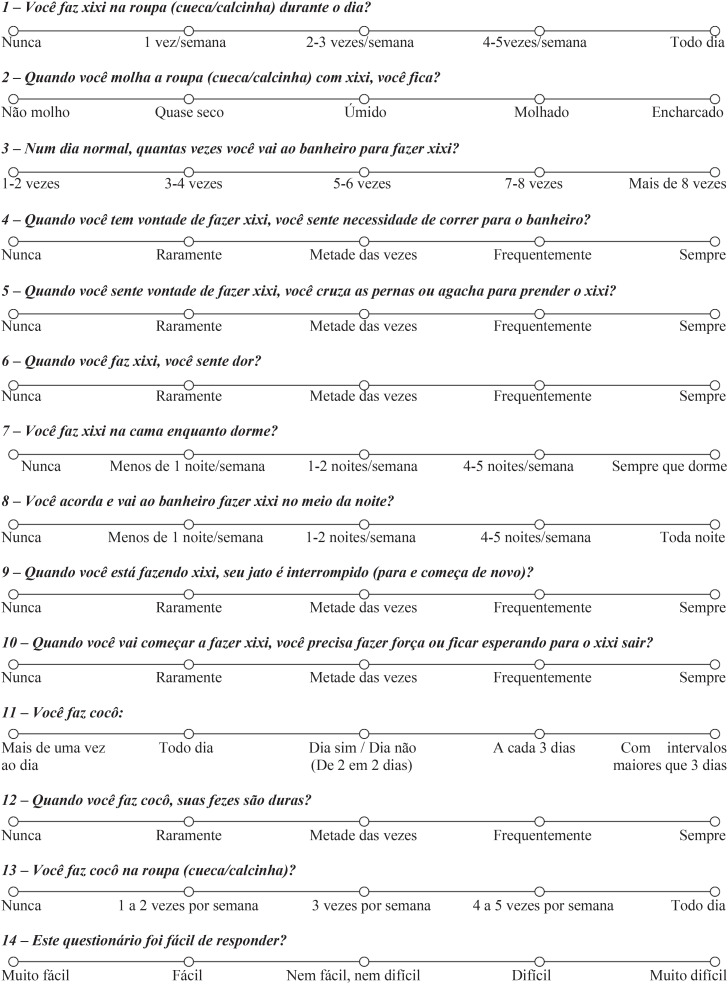
Brazilian version of the Vancouver Symptom Score (VSS).

A committee of experts analyzed the results of the pilot test. The necessary changes were made according to the difficulties encountered by the participants of the previous phase. After consensus, the final version nominated T5 of the questionnaire was prepared: the Brazilian version of the VSS (Figure-3).

#### Stage 2: Validation the Brazilian version of the VSS in a sample of Brazilian children and adolescents (15, 18–21)

### Study population

#### Inclusion criteria

The study group consisted of 126 consecutive children and adolescents with 5 to 16 years old, diagnosed with BBD, and who regularly attended a specialized LUTD outpatient clinic from January 2021 to July 2022. The control group (CG) was composed of children and adolescents without a diagnosis or symptoms of BBD from a local school randomly approached to participate in the study, matched by gender, age, and socioeconomic status with the cases.

#### Exclusion criteria

Children and adolescents with intellectual development disorders, congenital anomalies of the nervous system, urogenital malformations, presence of diseases and/or use of medications that interfere with the functioning of the bladder or urethral sphincter or that refused to participate in the study.

#### Sample Calculation

There is no consensus on the ideal sample size for validation studies. Terwee et al. (19) stated that criteria for the sample size needed for studies that assess measures were not defined. Rules-of-thumb vary from four to 10 subjects per variable, with a minimum number of 100 subjects (22).

#### Validation

Of the 126 study group participants, 19 refused to complete the questionnaires. The final sample was 107 patients with BBD. Similarly, 107 controls were invited to complete the questionnaire.

The parents (or caregivers) completed the questionnaire of children aged five to nine years old and adolescents aged 10 to 16 years old completed the questionnaire to measure completion times. A researcher used a stopwatch and recorded the time in minutes spent individually on the task. The completion of the questionnaire seven days after the first application assessed test-retest reliability.

For validation, the Brazilian version of the DVSS (13) was used (Supplementary Table-1). The cut-off values to indicate the presence of BBD were > six for females and > nine for males

### Statistical analysis

#### Psychometric properties

In accordance with the recommendations for the cross-cultural adaptation process, the following psychometric properties of the VSS questionnaire were evaluated in our study (15–19):

1. Reliability: In this study, measurement properties, internal consistency and test-retest reliability were evaluated. Internal consistency was evaluated by the Cronbach coefficient, when be greater than 0.7 indicates good internal consistency. A correlation above 0.7 in test-retest reliability indicates good internal consistency. Test-retest reliability was estimated by Pearson's correlation coefficient (19).
2. Validity: In this study, we evaluated the construct validity of measure and content properties. Pearson's correlation test compared the questionnaires VSS and DVSS.

Quantitative variables were expressed as medians and interquartile ranges, while qualitative variables were expressed as absolute values, percentages, or proportions. P values < 0.05 were considered statistically significant.

The software GraphPad Prism, version 9.0.3 (GraphPad Prism®, San Diego-CA, USA) was used for statistical analysis.

## RESULTS

The study group comprised 107 children and adolescents with the same number in control group. In both groups, parents or caregivers completely answered the VSS and DVSS questionnaires (Table-1).

**Table 1:** Sociodemographic characteristics of the children and adolescents with Bladder Bowel Dysfunction (BBD) and Control Group

Characteristics		Children and adolescentes with BBD (n=107)	Control group (n=107)	p-value
**Gender**				**0.12**
	Male	52.3 (56/107)	52.3 (56/107)	
	Female	47.7 (51/107)	47.7 (51/107)	
Mean Age years (SD)		9.2±2.84	9.6±2.98	0.09
Age Range years		(5.1-16)	(5.7-16)	
**Socioeconomic status**				**0.1**
	Categories A and B	36.4% (39/107)	43.9% (47/107)	
	Categories C, D and E	63.3% (68/107)	56.1% (60/107)	

BBD = Bladder bowel dysfunction; p value = Unpaired t test; SD = Standard deviation

**Figure 4:**
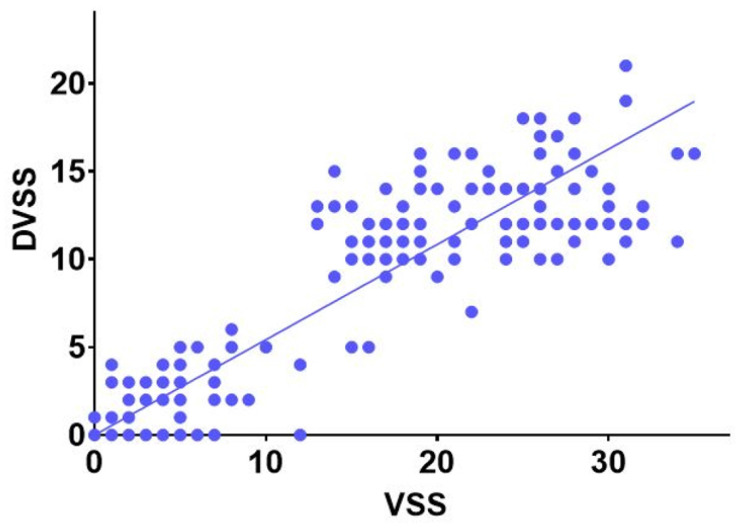
Correlation between total Vancouver Symptom Score (VSS) score and total Disfunction Voiding Symptom Score (DVSS) score.

There was a positive correlation (r = 0.91, 95% CI 0.88 to 0.93, p < 0.0001) between total VSS score and total DVSS score (Figure-4).

**Figure 5:**
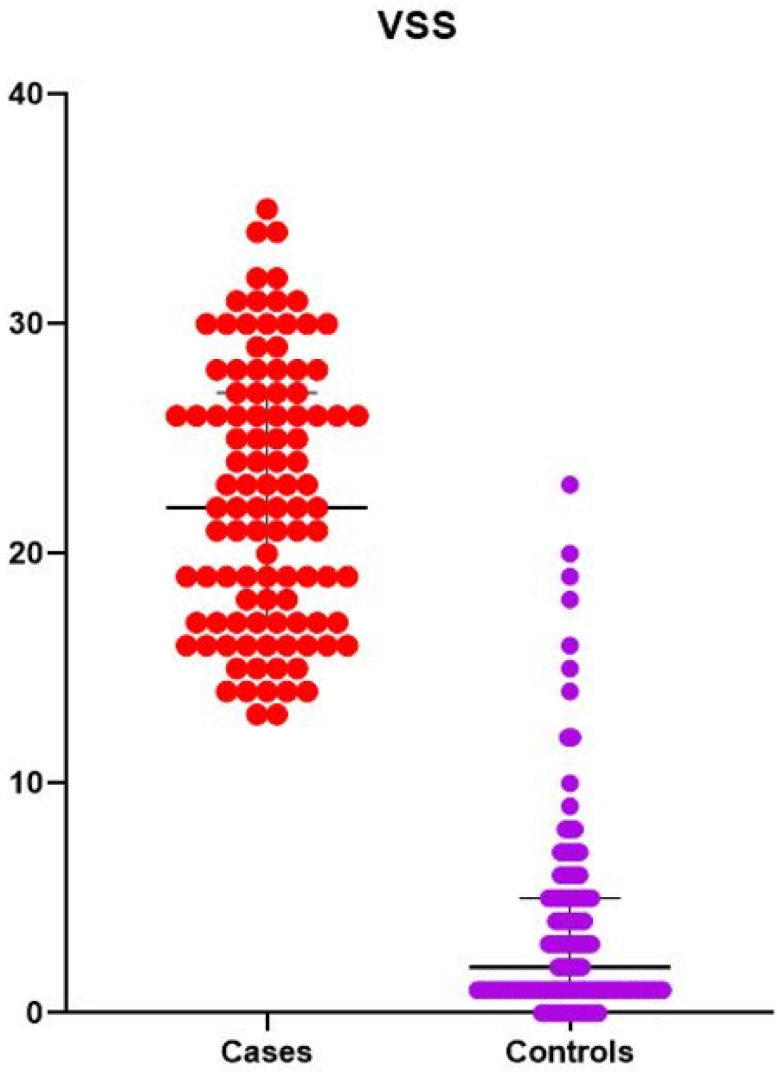
Median Vancouver Symptom Score (VSS) score in cases and controls.

The mean score for VSS in patients with BBD was 22, while in controls the same parameter was 2 (p < 0.0001) (Figure-5).

All participants (study and control group) rated the questionnaire as easy or very easy to answer. The mean time to complete the questionnaire was 3 minutes (ranging from two to six minutes).

The internal consistency estimated by Cronbach's alpha was 0.87 for BBD (95% lower confidence limit 0.85 to 0.82). We evaluated test-retest reliability in 97 cases and controls. The response rate was 93%, and the Pearson correlation coefficient was 0.94 (p<0.001), showing excellent reliability when the two questionnaires were answered one week apart.

**Figure 6:**
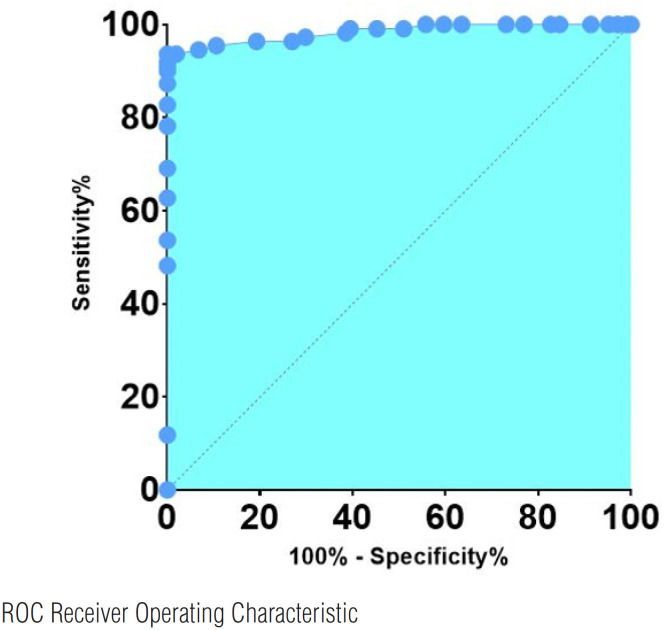
ROC curve for Vancouver Symptom Score (VSS).

VSS had excellent diagnostic accuracy in detecting BBD, with an area under the Receiver Operating Characteristic (ROC) curve (AUC) of 98% (95% CI: 0.96 to 0.99, p < 0.001) (Figure-6).

The cut-off value above 11 points yielded a sensitivity of 100% (95% CI 96.4% to 100%) and a specificity of 91.8% (95% CI 85.1% to 95.6%) with Likelihood ratio of 12.2. (Supplementary Table-2).

## DISCUSSION

This study reported the authorized translation, cross-cultural adaptation, and validation of the VSS to be used in Brazilian children and adolescents with BBD. The excellent comprehension values achieved during pre-test applications of the translated VSS in participants whose first language is Brazilian Portuguese showed that the translated scale was adequately adapted to the Brazilian culture. In addition, there were robust evidence of validity and reliability in this sample.

Translation and cultural adaptation are vital steps. In addition to language, cultural aspects considerably influence the understanding of an instrument. Therefore, the cross-cultural adaptation of the original components is necessary (15–18). The sequential phases of translation, back-translation and meetings between the translators and the team of specialists led to the development of descriptions adapted to a better understanding by the parents and Brazilian children and adolescents (15, 23, 24).

In the present study, a high correlation between VSS and DVSS was found. DVSS was chosen since it is one of the most used instruments for the evaluation of BBD symptoms and has already been validated in Brazilian population (13). We hypothesized that a significant correlation between VSS and DVSS would be found. Indeed, we detected a high positive correlation between the two questionnaires. Thus, data from the control group confirmed the potential of the VSS to differentiate between participants with and without symptoms of BBD. The BBD group had significantly higher scores than the control group, indicating a discriminative ability and possible diagnostic value of VSS for children and adolescents with BBD. In the analysis of internal consistency, a Cronbach's alpha of 0.87 in the patients’ group indicated good level of internal consistency. The value obtained was superior to the Cronbach's alpha (0.45) (9) described in the original validation study and the Dutch validation of the VSS (0.55) (11).

Excellent test-retest reliability was also found (Pearson coefficient 0.94), similar to the original study (0.89) (9) and better than the Dutch VSS validation study (0.41) (11). We chose a week to repeat the test. In line with the original research, it is unlikely that the symptoms of BBD will change in such a short amount of time, even though we have initiated or prescribed treatment modifications. Furthermore, repeated responses were rarely based solely on recall of the first questionnaire (9). The retest in the Dutch study was carried out within 15 days, and they made general recommendations related to voiding and bowel function at baseline. Thus, the authors found that this determined some improvement in BBD symptoms within 15 days. Therefore, by selecting only patients whose test-retest period was one week, the Dutch version of the VSS showed adequate reliability and test-retest (0.79- 0.94) (11).

In our study, the VSS had excellent diagnostic accuracy in detecting patients with BBD, with an AUC of 98%. A cut-off scores above 11 points had a diagnostic sensitivity of 100% and a specificity of 91.61%. This finding is supported by the study of Asfhar et al., (9) in which the AUC was of 98%. The authors also showed that a score of 11 had diagnostic sensitivity and specificity of 80% and 91%, respectively. The high sensitivity and specificity of the VSS questionnaire indicate that this instrument is a valuable screening tool, since it allows the early identification and referral of children and adolescents with symptoms of BBD to specialized centers (3, 5, 7, 9–11). The early diagnosis and adequate multidisciplinary treatment can avoid the repercussions for the upper urinary tract mainly represented by renal scars (5). Additionally, the treatment can improve self-esteem and the quality of family, school, and social life (6).

All participants with BBD and controls rated the questionnaire as easy or very easy to answer and took a few minutes to complete the questionnaire. These data are similar to those obtained in the study of Asfhar et al. (9) in which 85% of the participants classified the questionnaire as easy or very easy to answer.

The major limitation of the present study is that the VSS was not tested to detect changes after different treatments, that is, an analysis of responsiveness. Future research should focus on the responsiveness and clinical applicability of the Brazilian version of the VSS.

BBD is a common condition in the urology practice and often appears subjective to the attending physician's judgment. The lack of a validated instrument to diagnose this condition negatively impacts clinical practice and research, creating a heterogeneous patient population across different studies and, consequently, inconsistent results. We have chosen the VSS because this instrument is short, easy to apply, well established in the literature for the diagnose of BBD, and superior to other scores used in our population. The validation of VSS is an essential issue to use this questionnaire in Brazilian population.

## CONCLUSION

The Vancouver Symptom Score translated, cross-culturally adapted, and validated for the Brazilian population seems to be a reliable and valid tool to identify symptoms of bladder bowel dysfunction in children and adolescents aged five to 16 years whose first language is Brazilian Portuguese. The authors believe that this version will be helpful for clinical practice and scientific research in Brazil.

## ABBREVIATIONS

AUC: Area under the ROC Curve
BBD: Bladder bowel dysfunction
CBBDQ: Childhood Bladder and Bowel Dysfunction Questionnaire
DVSS: Dysfunctional Voiding Symptom Score
LUTD: Lower urinary tract dysfunction (LUTD)
VSS: Vancouver Symptom Score
ROC: Receiver Operating Characteristic (ROC)

## APPENDIX

**Supplementary table 1:** Brazilian version of the Dysfunctional Voiding Symptom Score (DVSS) adapted from Calado et al. 2010 (13).

Durante os últimos 30 dias		Nunca ou Quase nunca	Menos Que Metade do Tempo	A Metade do tempo	Quase Todo o Tempo
1.	Seu(a) filho(a)tem molhado de xixi a roupa durante o dia?	0	1	2	3
2.	Quando seu(a) filho(a) se molha de xixi, a cueca ou calcinha fica ensopada?	0	1	2	3
3.	Com que frequência seu(a) filho(a) não faz cocô todos os dias?	0	1	2	3
4.	Seu(a) filho(a) tem que fazer força para fazer cocô?	0	1	2	3
5.	Com que frequência seu(a) filho(a) só vai ao banheiro fazer xixi uma ou duas vezes por dia?	0	1	2	3
6.	Seu(a) filho(a) segura o xixi cruzando as pernas, agachando ou dançando?	0	1	2	3
7.	Quando seu(a) filho(a) precisa fazer xixi tem que ir rápido ao banheiro? (não consegue esperar)	0	1	2	3
8.	Seu(a) filho(a) tem que fazer força para fazer xixi?	0	1	2	3
9.	Seu(a) filho(a) disse que sente dor quando faz xixi?	0	1	2	3
10.	10. Seu(a) filho(a) passou por alguma situação estressante como as dos exemplos abaixo nos últimos 30 dias? Marque ao lado sim ou não.				
•	Bebê novo em casa				
•	Mudança de casa				
•	Mudança de escola				
•	Problemas escolares				
•	Abuso(sexual/físico)	Não (0)		Sim (3)	
•	Problemas em casa (divórcio/morte)				
•	Eventos especiais (aniversário)				
•	Acidente/ferimento				
•	Outros				

**Supplementary Table-2:** Details of the specificity and sensitivity of the Vancouver Symptom Score (VSS) scores.

	Sensitivity%	95% CI	Specificity%	95% CI	Likelihood ratio
> 0.5000	100.0	96.44% to 100.0%	11.82	7.038% to 19.18%	1.134
> 1.500	100.0	96.44% to 100.0%	48.18	39.06% to 57.42%	1.930
> 2.500	100.0	96.44% to 100.0%	53.64	44.35% to 62.67%	2.157
> 3.500	100.0	96.44% to 100.0%	62.73	53.41% to 71.19%	2.683
> 4.500	100.0	96.44% to 100.0%	69.09	59.93% to 76.96%	3.235
> 5.500	100.0	96.44% to 100.0%	78.18	69.58% to 84.88%	4.583
> 6.500	100.0	96.44% to 100.0%	82.73	74.59% to 88.65%	5.789
> 7.500	100.0	96.44% to 100.0%	87.27	79.76% to 92.27%	7.857
> 8.500	100.0	96.44% to 100.0%	90.00	82.98% to 94.32%	10.00
> 9.500	100.0	96.44% to 100.0%	90.91	84.07% to 94.99%	11.00
> 11.00	100.0	96.44% to 100.0%	91.82	85.18% to 95.64%	12.22
> 12.50	100.0	96.44% to 100.0%	93.64	87.44% to 96.88%	15.71
> 13.50	98.08	93.26% to 99.66%	93.64	87.44% to 96.88%	15.41
> 14.50	93.27	86.75% to 96.70%	94.55	88.61% to 97.48%	17.10
> 15.50	89.42	82.05% to 93.99%	95.45	89.80% to 98.04%	19.67
> 16.50	80.77	72.15% to 87.19%	96.36	91.02% to 98.58%	22.21
> 17.50	73.08	63.84% to 80.67%	96.36	91.02% to 98.58%	20.10
> 18.50	70.19	60.81% to 78.14%	97.27	92.29% to 99.26%	25.74
> 19.50	61.54	51.94% to 70.32%	98.18	93.61% to 99.68%	33.85
> 20.50	60.58	50.97% to 69.43%	99.09	95.03% to 99.95%	66.63
> 21.50	54.81	45.24% to 64.03%	99.09	95.03% to 99.95%	60.29
> 22.50	49.04	39.64% to 58.51%	99.09	95.03% to 99.95%	53.94

CI = Confidence Interval
